# Supplementary figures and images for: Categorical encoding of decision variables in orbitofrontal cortex
Source: PLoS Comput Biol. 2019 Oct 14;15(10):e1006667. doi: 10.1371/journal.pcbi.1006667 (PMC6812845; doi:10.1371/journal.pcbi.1006667)

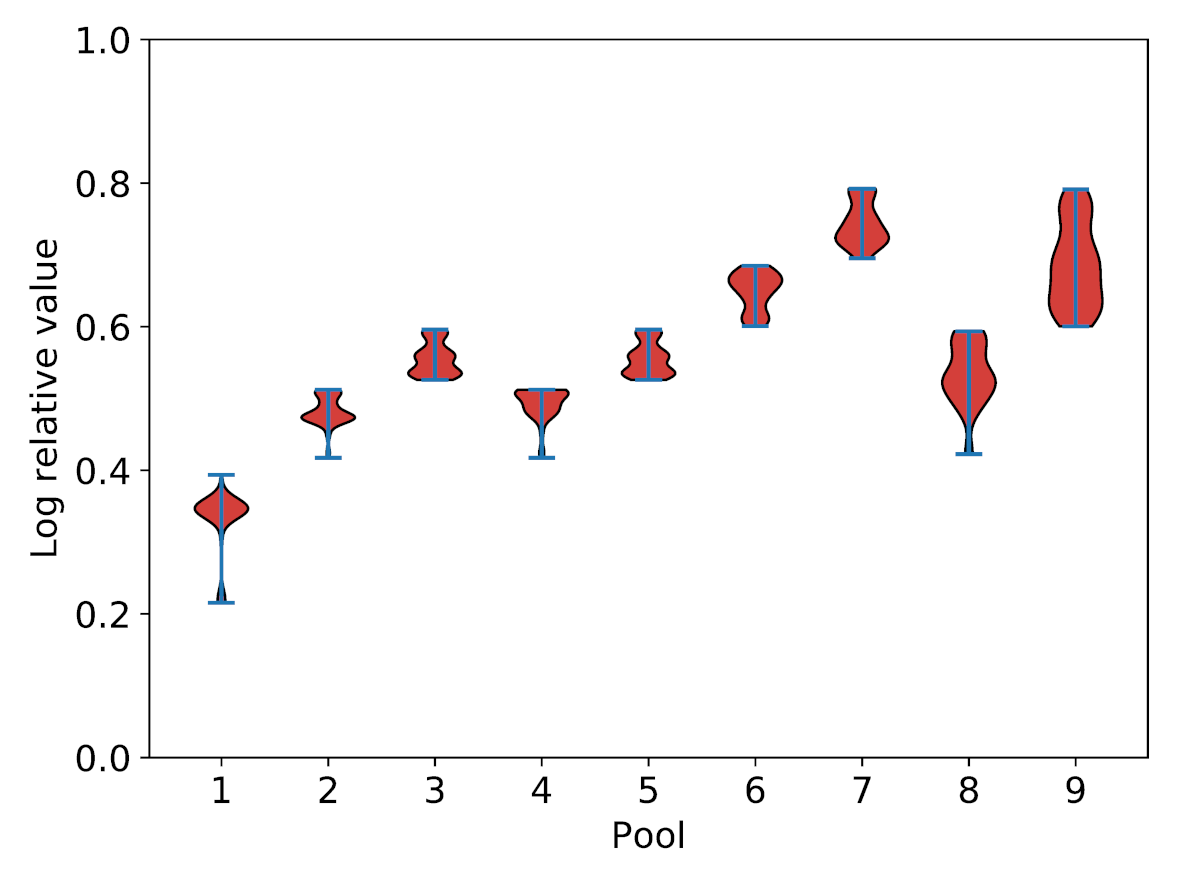

Supplement: S1 Fig — Violin plots of the distributions of relative values. The values were log transformed for better visibility. Each component shows the distribution for one pool. The ranges of distribution support indicate that the relative values are similar in each pool. (TIF) [file pcbi.1006667.s001.tif]

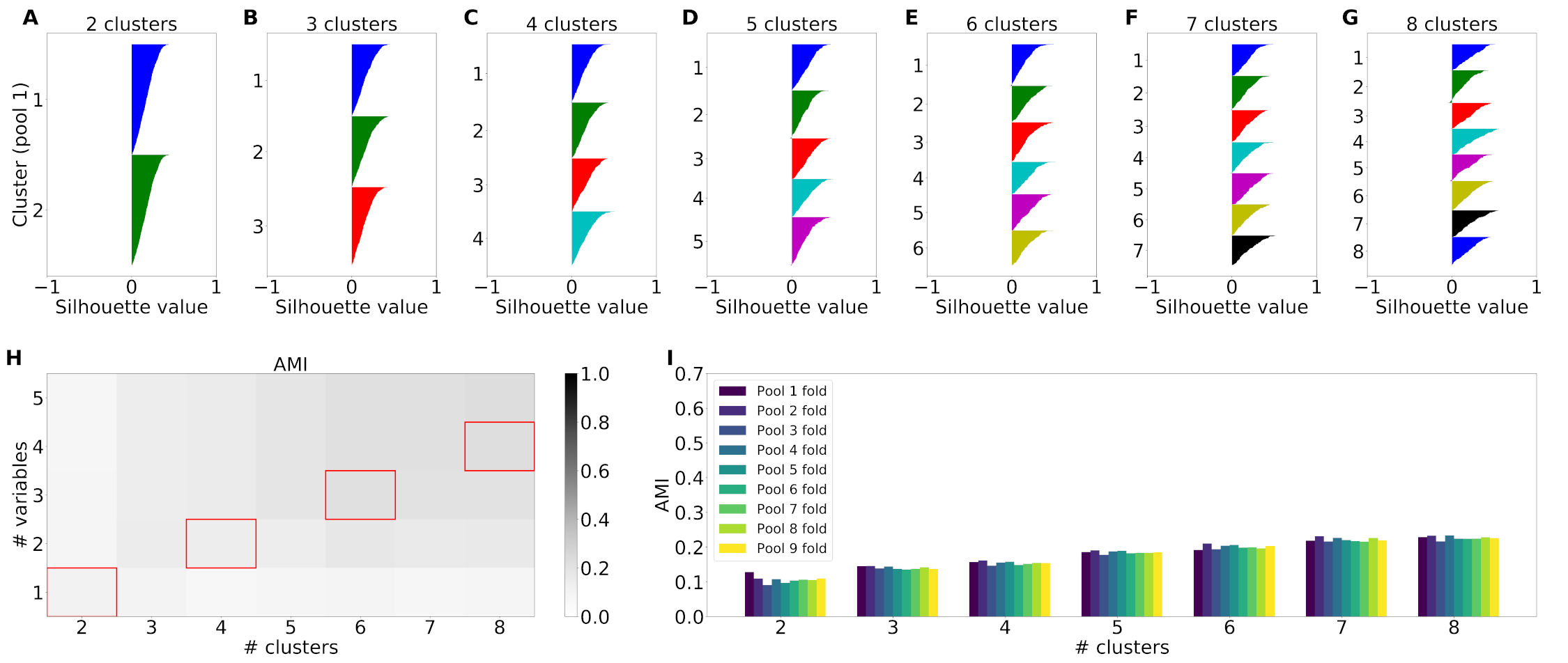

Supplement: S2 Fig — Within each pool and each trial type, neural responses were randomly shuffled to destroy categories but preserve response distributions within each trial type. (A)-(F) Silhouette plots for the spherical k-means partitions of one example pool. Each color corresponds to one cluster. The number of clusters was varied between 2 (A) and 8 (G). (H) Adjusted mutual information cluster similarity between spherical k-means clustering and variable-based centroid clustering as a function of the number of clusters and number of variables over all pools. Corresponding numbers of clusters and numbers of variables are marked in red. (I) Maximum adjusted mutual information for each number of clusters with Jackknife estimated standard errors. (TIF) [file pcbi.1006667.s002.tif]

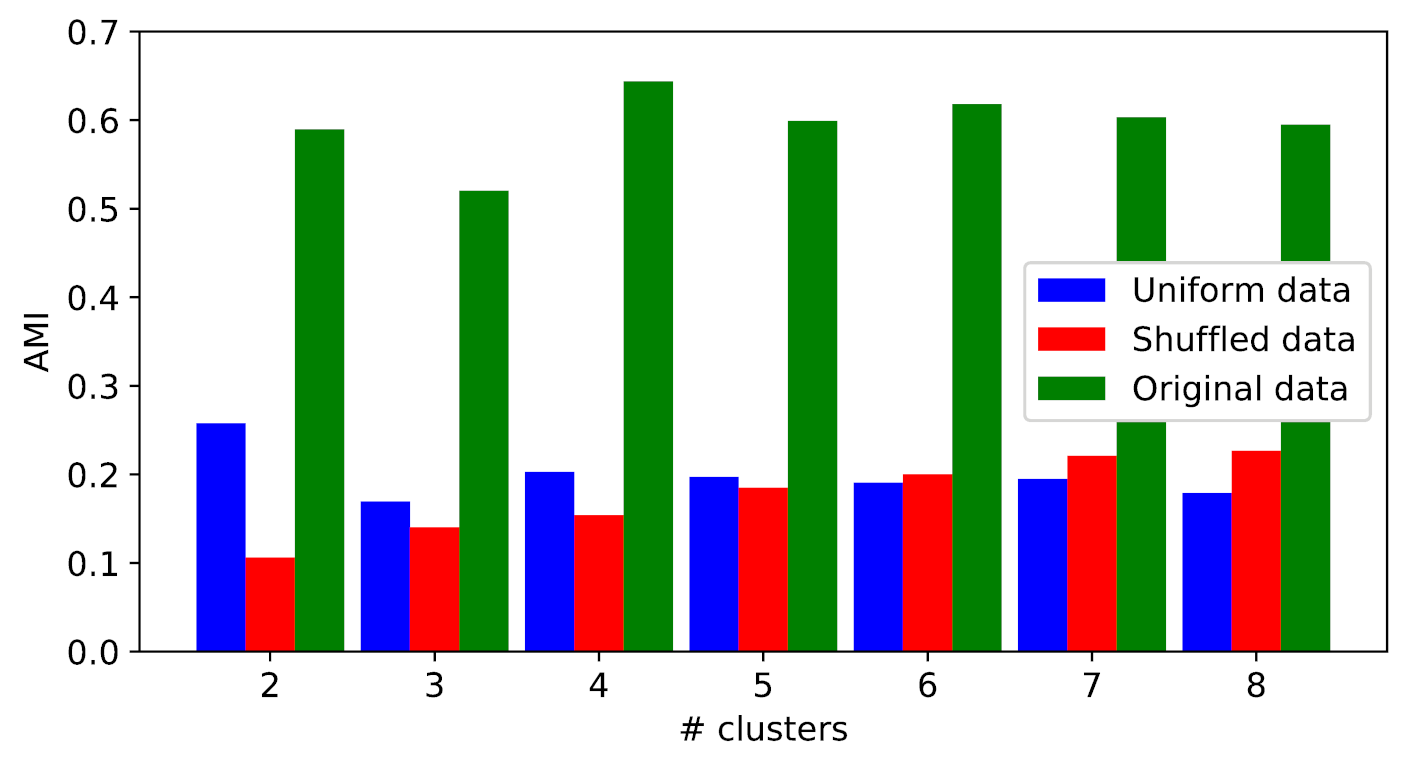

Supplement: S3 Fig — Maximum adjusted mutual information over number of variables for different numbers of clusters as in Fig 6F (“Uniform data”), S2 Fig I collapsed over folds (“Shuffled data”) and Fig 7I collapsed over folds (“Original data”). (TIF) [file pcbi.1006667.s003.tif]

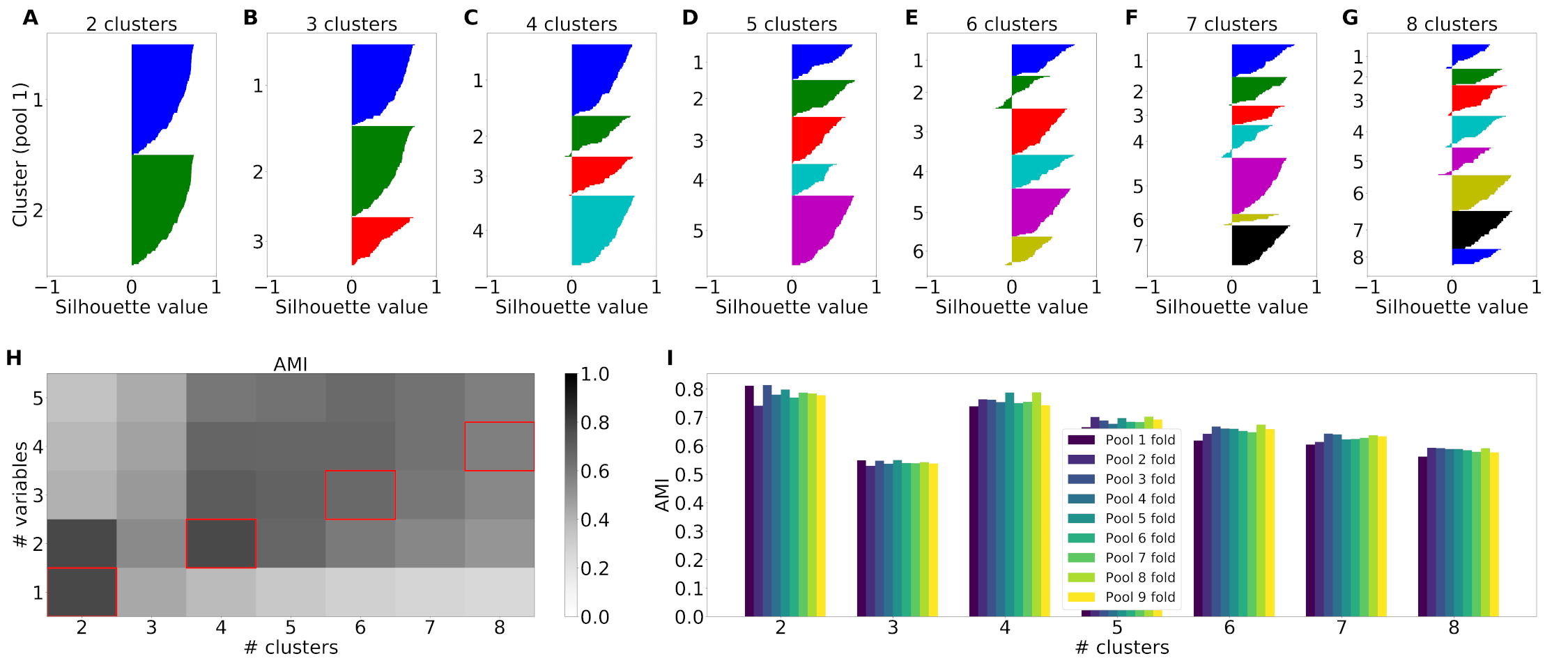

Supplement: S4 Fig — In this analysis, neural responses were taken from the post-offer time window only. (A)-(G) Silhouette plots for the spherical k-means partitions of one example pool. Each color corresponds to one cluster. The number of clusters was varied between 2 (A) and 8 (G). (H) Adjusted mutual information cluster similarity between spherical k-means clustering and variable-based centroid clustering as a function of the number of clusters and number of variables over all pools. Corresponding numbers of clusters and numbers of variables are marked in red. (I) Maximum adjusted mutual information for each number of clusters where each bar shows the result of one Jackknife fold. (TIF) [file pcbi.1006667.s004.tif]

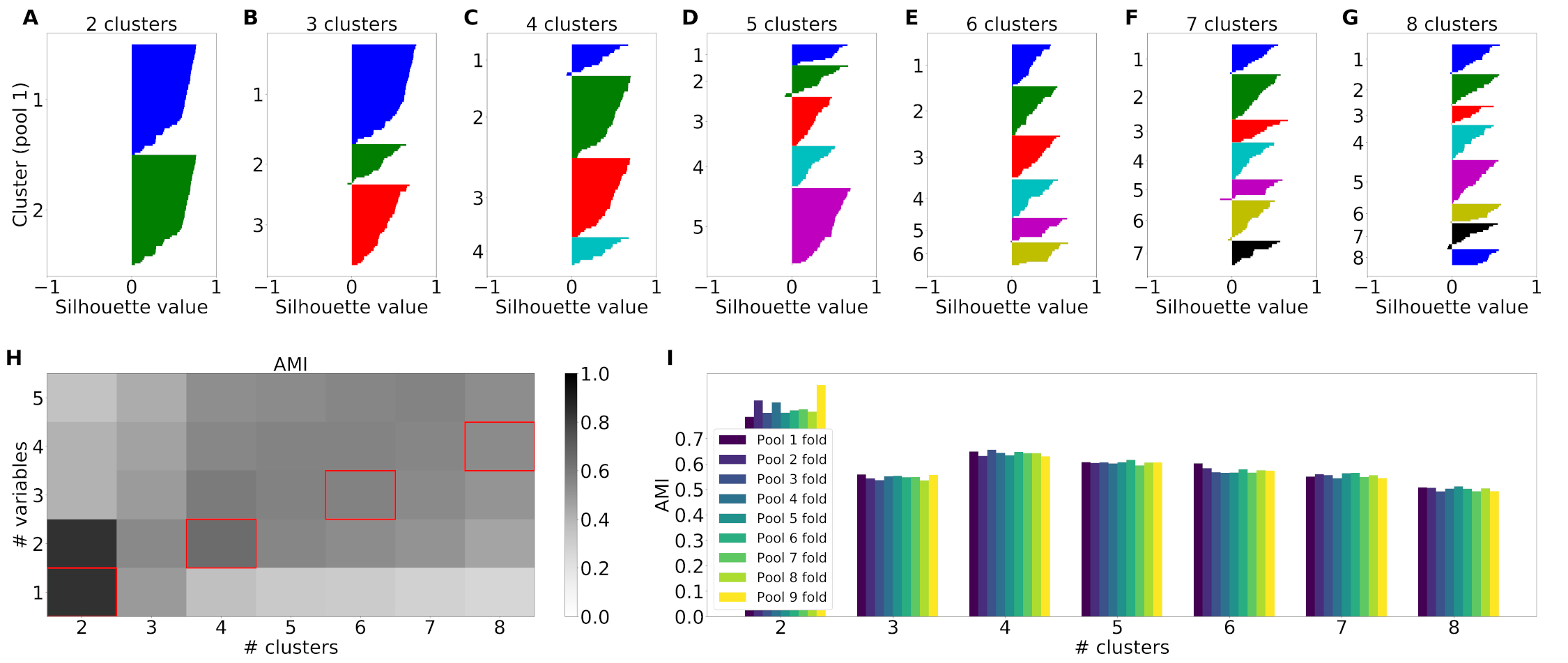

Supplement: S5 Fig — In this analysis, neural responses were taken from the late-delay time window only. (A)-(G) Silhouette plots for the spherical k-means partitions of one example pool. Each color corresponds to one cluster. The number of clusters was varied between 2 (A) and 8 (G). (H) Adjusted mutual information cluster similarity between spherical k-means clustering and variable-based centroid clustering as a function of the number of clusters and number of variables over all pools. Corresponding numbers of clusters and numbers of variables are marked in red. (I) Maximum adjusted mutual information for each number of clusters where each bar shows the result of one Jackknife fold. (TIF) [file pcbi.1006667.s005.tif]

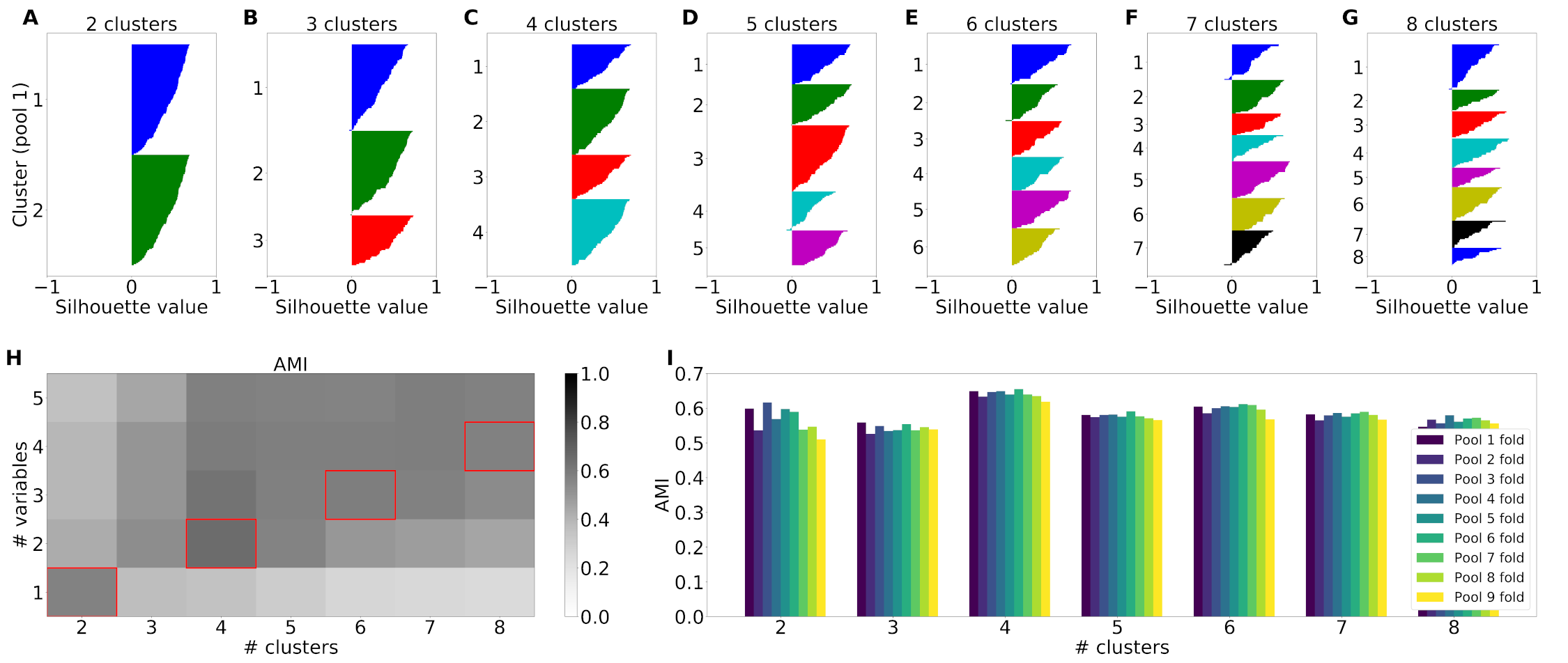

Supplement: S6 Fig — In this analysis, neural responses were taken from the pre-juice time window only. (A)-(G) Silhouette plots for the spherical k-means partitions of one example pool. Each color corresponds to one cluster. The number of clusters was varied between 2 (A) and 8 (G). (H) Adjusted mutual information cluster similarity between spherical k-means clustering and variable-based centroid clustering as a function of the number of clusters and number of variables over all pools. Corresponding numbers of clusters and numbers of variables are marked in red. (I) Maximum adjusted mutual information for each number of clusters where each bar shows the result of one Jackknife fold. (TIF) [file pcbi.1006667.s006.tif]

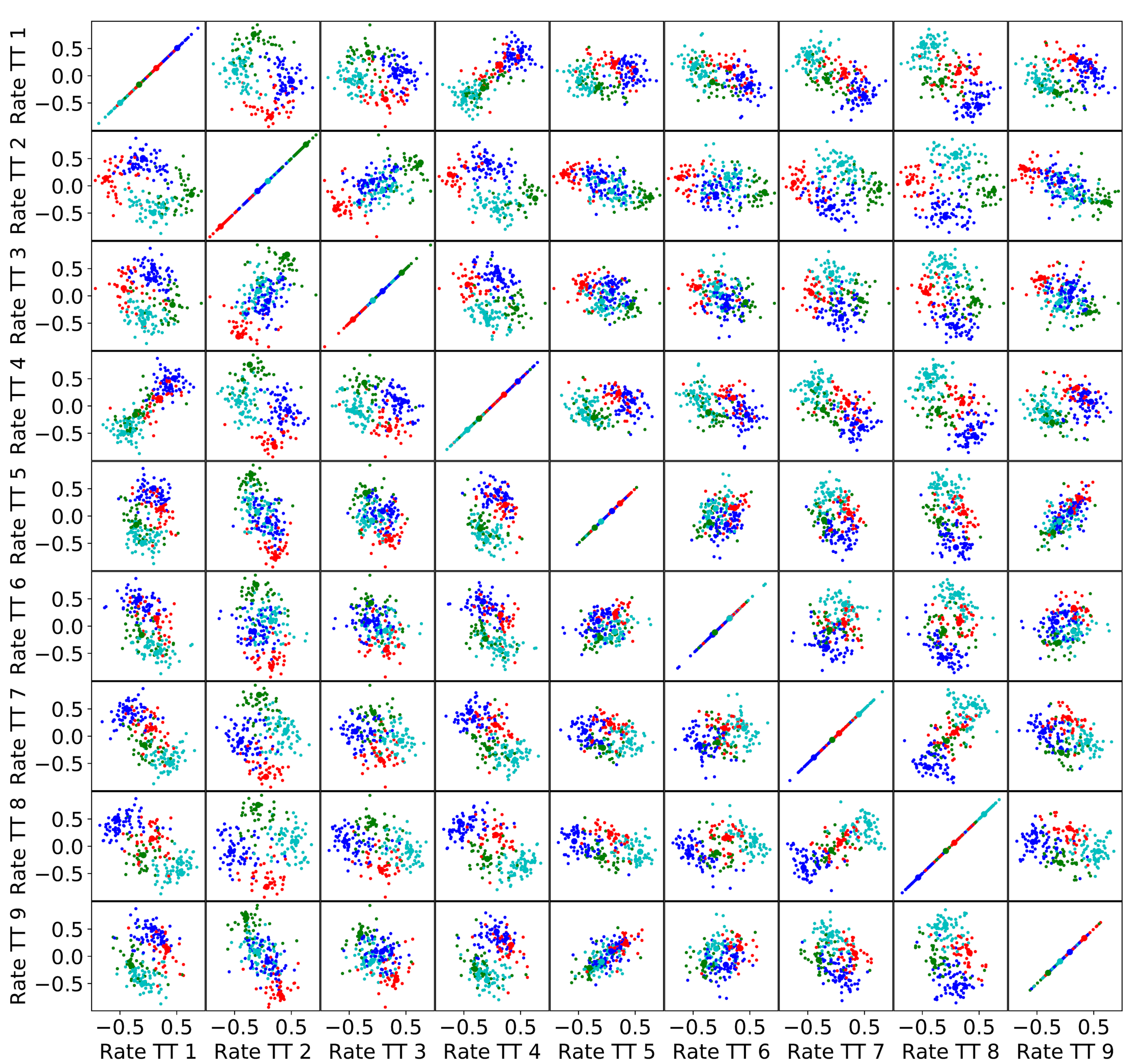

Supplement: S7 Fig — Like Fig 8 but neural responses were taken from the post-offer time window only. Each color corresponds to one cluster. Each panel shows the centered and normalized firing rates of a pair of trial types and each point in a panel represents a cell from pool 1. Cluster centers are marked with black circles. (TIF) [file pcbi.1006667.s007.tif]

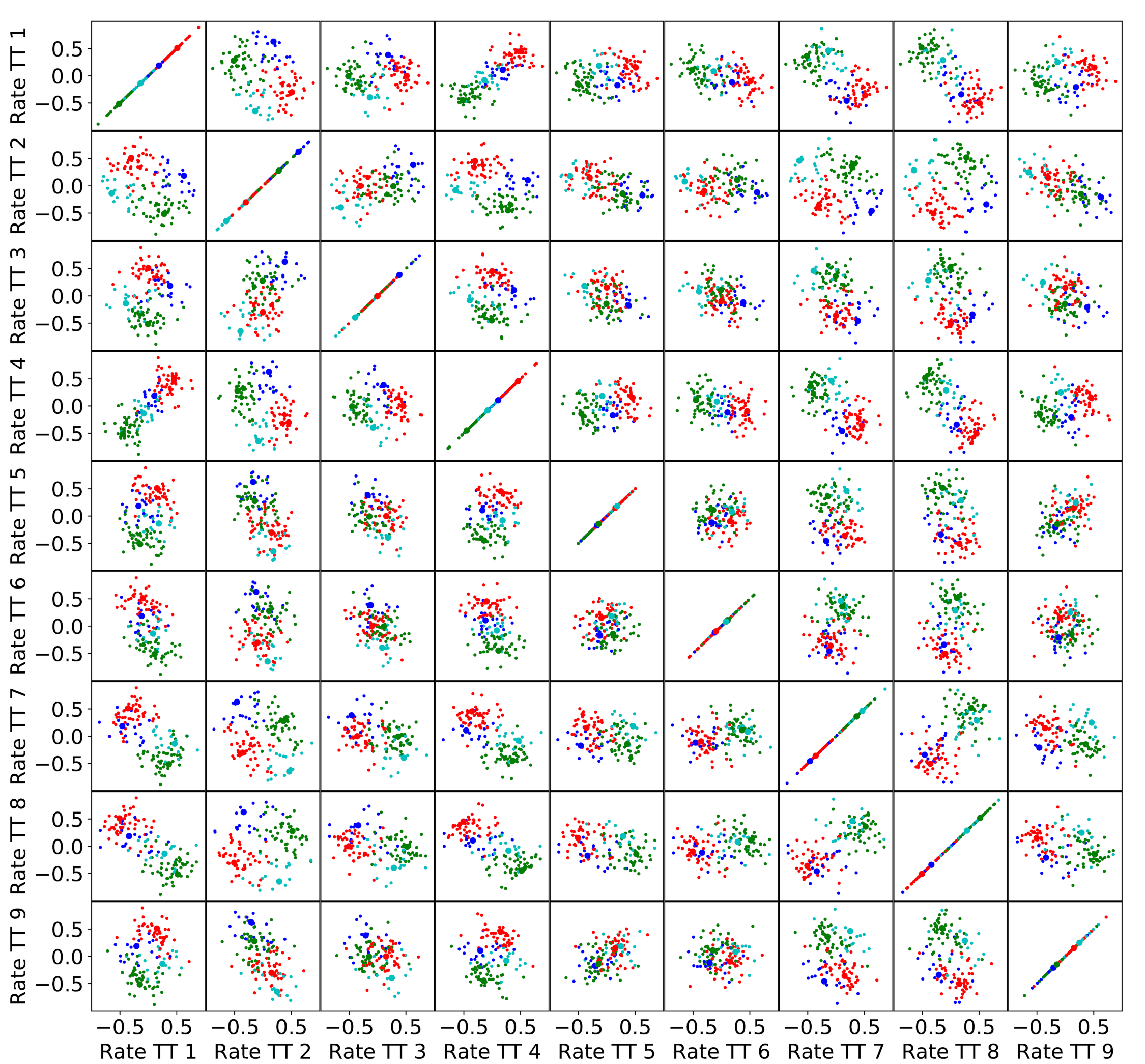

Supplement: S8 Fig — Like Fig 8 but neural responses were taken from the late-delay time window only. Each color corresponds to one cluster. Each panel shows the centered and normalized firing rates of a pair of trial types and each point in a panel represents a cell from pool 1. Cluster centers are marked with black circles. (TIF) [file pcbi.1006667.s008.tif]

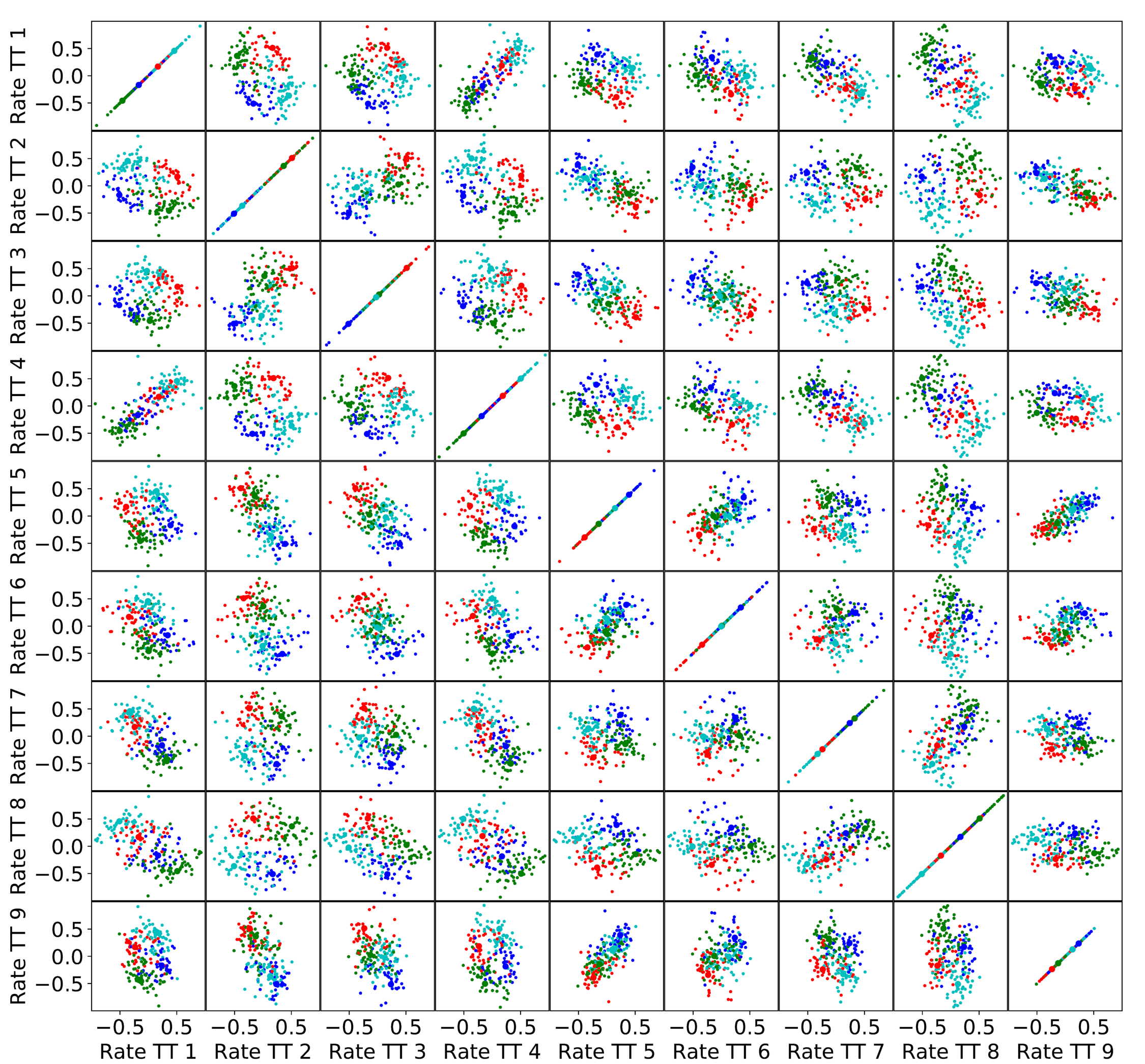

Supplement: S9 Fig — Like Fig 8 but neural responses were taken from the pre-juice time window only. Each color corresponds to one cluster. Each panel shows the centered and normalized firing rates of a pair of trial types and each point in a panel represents a cell from pool 1. Cluster centers are marked with black circles. (TIF) [file pcbi.1006667.s009.tif]

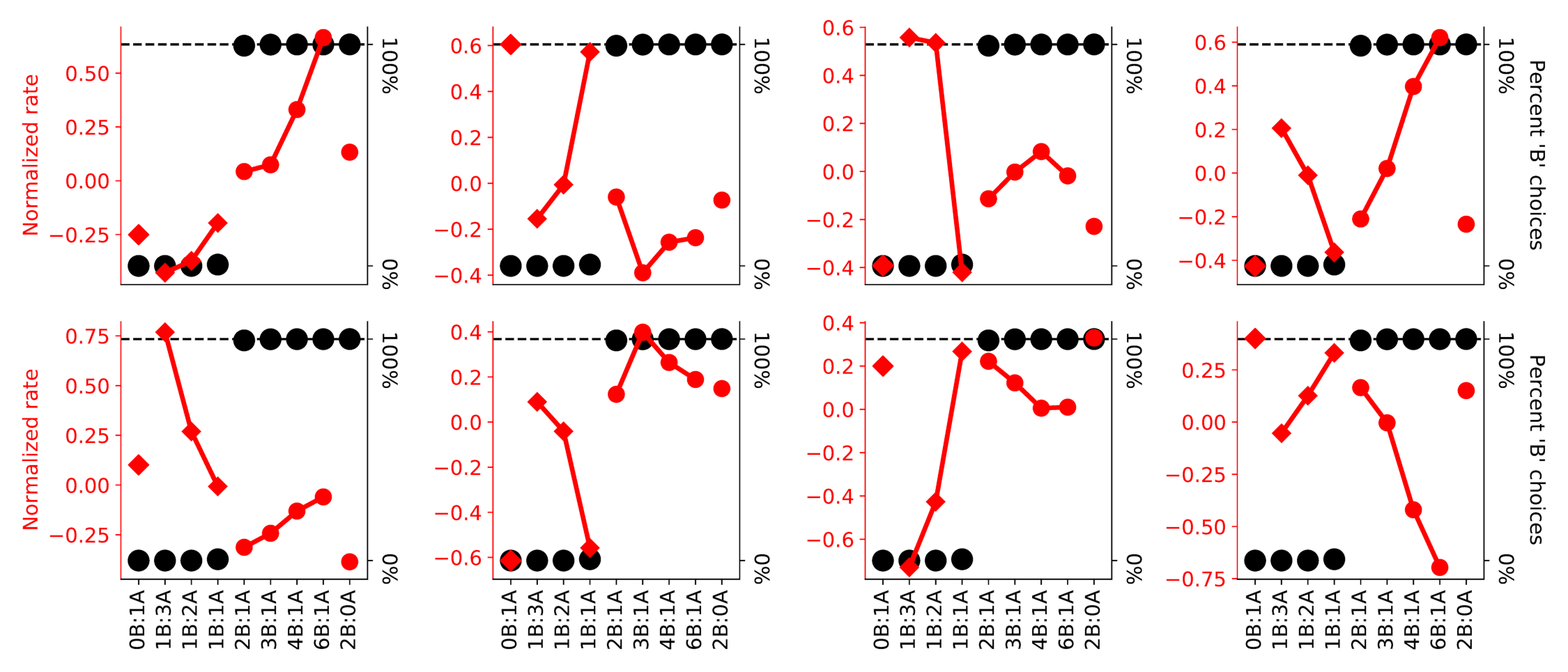

Supplement: S10 Fig — Like Fig 9 but neural responses were taken from the post-offer time window only. The number of clusters is lower than for the full data set (6 clusters instead of 8 clusters). The x-axis represents offer types ranked by the ratio #B:#A. The y-axis in red represents normalized response rates. The y-axis in black shows monkey behavior. Red diamonds represent the responses to chosen juice A whereas red dots represent the responses to chosen juice B. The separate red diamond and red dot show forced choices. (TIF) [file pcbi.1006667.s010.tif]

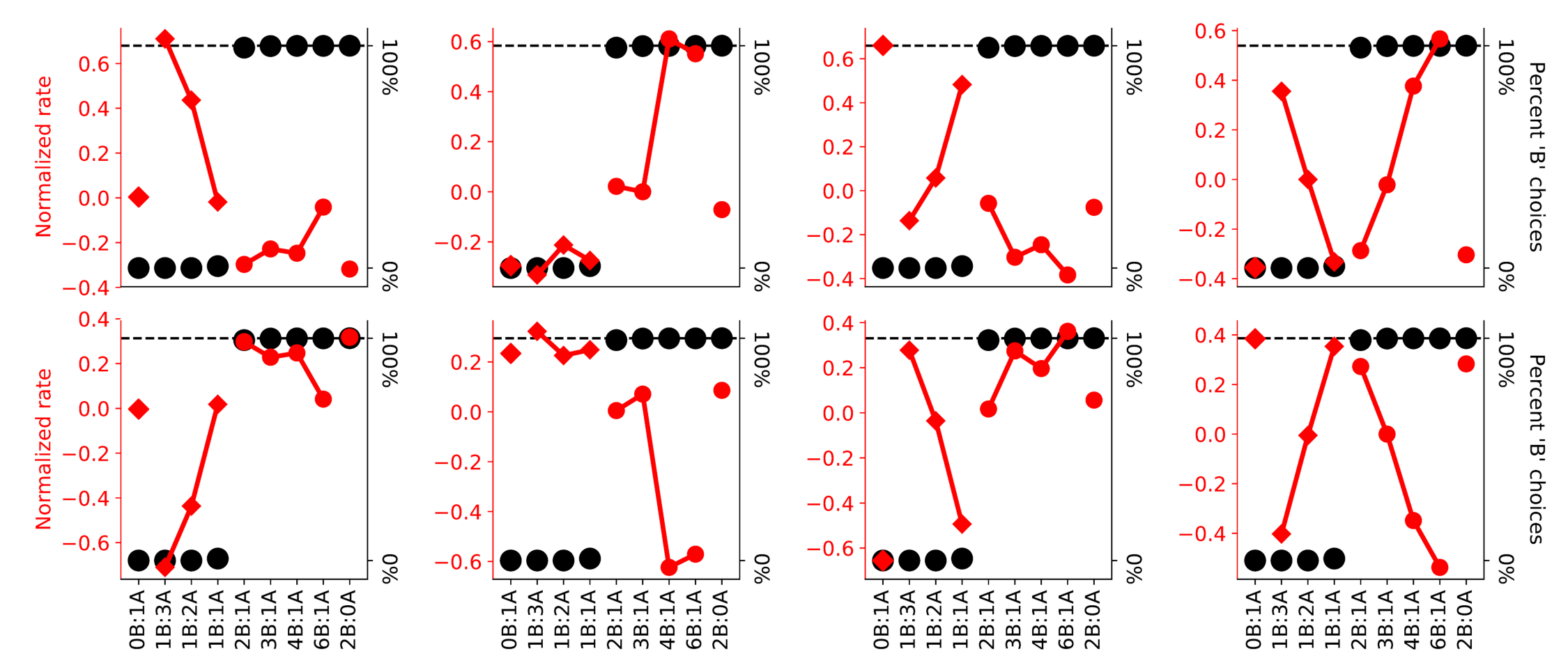

Supplement: S11 Fig — Like Fig 9 but neural responses were taken from the late-delay time window only. The number of clusters is the same as for the full data set. The x-axis represents offer types ranked by the ratio #B:#A. The y-axis in red represents normalized response rates. The y-axis in black shows monkey behavior. Red diamonds represent the responses to chosen juice A whereas red dots represent the responses to chosen juice B. The separate red diamond and red dot show forced choices. (TIF) [file pcbi.1006667.s011.tif]

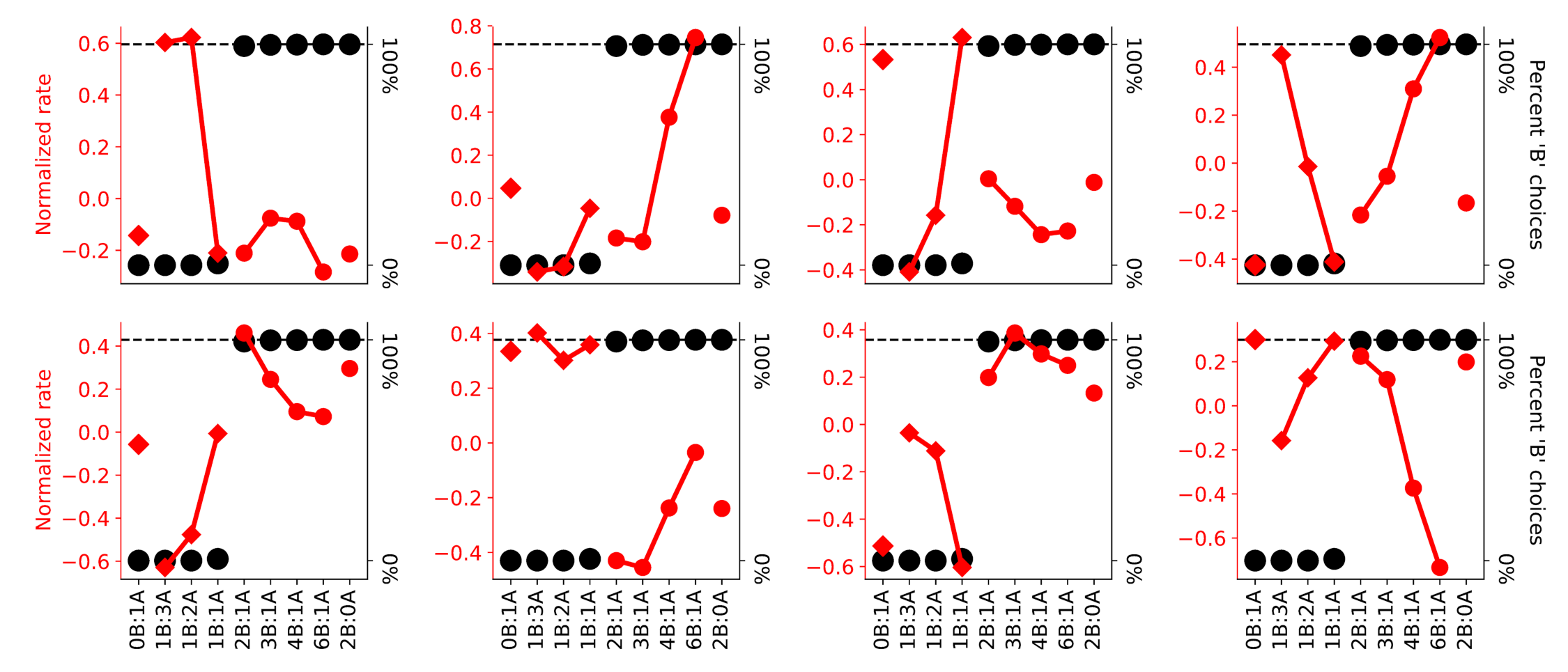

Supplement: S12 Fig — Like Fig 9 but neural responses were taken from the pre-juice time window only. The number of clusters is the same as for the full data set. The x-axis represents offer types ranked by the ratio #B:#A. The y-axis in red represents normalized response rates. The y-axis in black shows monkey behavior. Red diamonds represent the responses to chosen juice A whereas red dots represent the responses to chosen juice B. The separate red diamond and red dot show forced choices. (TIF) [file pcbi.1006667.s012.tif]

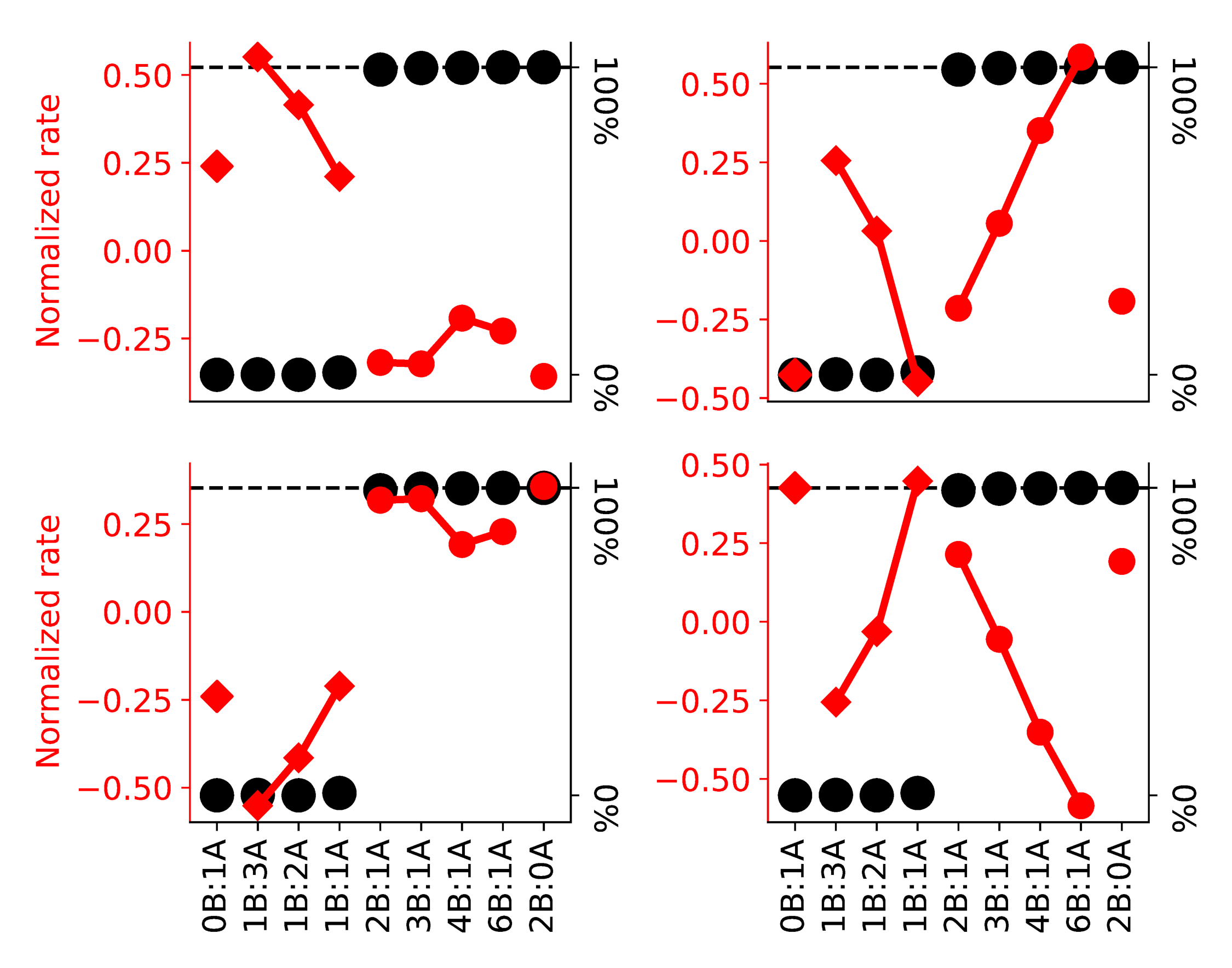

Supplement: S13 Fig — Like Fig 9 but for 4 cluster and 2 variables. The x-axis represents offer types ranked by the ratio #B:#A. The y-axis in red represents normalized response rates. The y-axis in black shows monkey behavior. Red diamonds represent the responses to chosen juice A whereas red dots represent the responses to chosen juice B. The separate red diamond and red dot show forced choices. (TIF) [file pcbi.1006667.s013.tif]
